# *Centella asiatica*: Advances in Extraction Technologies, Phytochemistry, and Therapeutic Applications

**DOI:** 10.3390/life15071081

**Published:** 2025-07-09

**Authors:** Zaw Myo Hein, Prarthana Kalerammana Gopalakrishna, Anil Kumar Kanuri, Warren Thomas, Farida Hussan, Venkatesh R. Naik, Nisha Shantakumari, Muhammad Danial Che Ramli, Mohamad Aris Mohd Moklas, Che Mohd Nasril Che Mohd Nassir, Thirupathirao Vishnumukkala

**Affiliations:** 1 Department of Basic Medical Sciences, College of Medicine, Ajman University, Ajman P.O. BOX 346, United Arab Emirates; z.hein@ajman.ac.ae (Z.M.H.); n.kumari@ajman.ac.ae (N.S.); 2 Physiology Discipline, Human Biology Division, School of Medicine, IMU University, Kuala Lumpur 57000, Malaysia; prarthana@imu.edu.my; 3 Department of Pharmaceutical Technology, Faculty of Pharmacy, Universiti Malaya, Kuala Lumpur 50603, Malaysia; anilkumarkanuri@um.edu.my; 4 Department of Human Biology, Royal College of Surgeons in Ireland, Medical University of Bahrain, Al Sayh P.O. Box 15503, Bahrain; 5 Anatomy Discipline, Human Biology Division, School of Medicine, IMU University, Kuala Lumpur 57000, Malaysia; faridahussan@imu.edu.my; 6 Department of Preclinical Sciences, Faculty of Medicine and Health Sciences, Universiti Tunku Abdul Rahman, Kajang 43000, Malaysia; venkatesh@utar.edu.my; 7 Department of Diagnostic and Allied Health Science, Faculty of Health and Life Sciences, Management and Science University, Shah Alam 40100, Malaysia; muhddanial_cheramli@msu.edu.my; 8 Department of Human Anatomy, Faculty of Medicine and Health Sciences, Universiti Putra Malaysia, Serdang 43400, Malaysia; aris@upm.edu.my; 9 Department of Anatomy and Physiology, Faculty of Medicine, Universiti Sultan Zainal Abidin, Kuala Terengganu 20400, Malaysia

**Keywords:** *Centella asiatica*, triterpenoids, analytical, quality control, extraction

## Abstract

*Centella asiatica* (*C. asiatica*) has attracted significant scientific interest due to its extensive medicinal properties and long-established use in traditional medicine. This review synthesizes recent advances in the technological exploitation of *C. asiatica*, covering the extraction of bioactive constituents to product development. Modern extraction techniques such as supercritical fluid extraction (SFE) and microwave-assisted extraction (MAE) have substantially improved the yield, selectivity, and preservation of key phytochemicals, particularly triterpenoids, saponins, and flavonoids. These compounds are now routinely characterized using advanced analytical platforms, ensuring product quality, consistency, and standardization. Moreover, the use of innovative formulation technologies and advanced delivery systems has facilitated the development of *C. asiatica*-based products tailored for various therapeutic areas, including pharmaceuticals, nutraceuticals, and cosmeceuticals targeting neuroprotection, wound healing, skin aging, and stress modulation. Alongside these developments, stringent quality control protocols, toxicological evaluations, and adherence to evolving regulatory standards enhance the safety and efficacy of *C. asiatica*-derived interventions. This review highlights the integration of traditional knowledge with modern science across the domains of extraction, analysis, formulation, and regulation. It serves as a comprehensive resource for researchers, formulators, and regulatory stakeholders aiming to develop high-quality, evidence-based *C. asiatica* products with improved bioavailability and therapeutic value.

## 1. Introduction

*Centella asiatica* (*C. asiatica*), commonly known as Gotu Kola or Pegaga, belonging to the Apiaceae family, is a perennial herbaceous plant renowned for its extensive use in traditional medicine, such as Ayurveda, traditional Chinese medicine, and Southeast Asian ethnomedicine [1]. Renowned for its cognitive-enhancing, wound-healing, anti-inflammatory, and adaptogenic properties, *C. asiatica* has become a focal point of modern phytopharmacological research [2]. This plant, rich in triterpenoids, saponins, flavonoids, and essential oil, continues to inspire the development of evidence-based herbal therapeutics [3].

With the global resurgence in interest toward plant-based medicines and natural health products, *C. asiatica* has emerged as a model species bridging traditional use and modern scientific validation. However, despite centuries of anecdotal evidence supporting its efficacy, translating this botanical legacy into standardized, clinically effective, and regulatory-compliant health products remains a formidable challenge [4]. This review explores the evolving landscape of *C. asiatica* research and development, emphasizing recent advances in extraction, phytochemical profiling, formulation science, therapeutic application, and regulatory considerations.

One of the most transformative aspects of harnessing *C. asiatica*’s potential lies in the advancement of extraction technologies. Traditional solvent-based methods, though effective, often suffer from limitations including low selectivity, long extraction times, and degradation of thermolabile compounds [5,6]. In response, cutting-edge methods such as supercritical fluid extraction (SFE), microwave-assisted extraction (MAE), and ultrasound-assisted extraction (UAE) have been developed [6]. These techniques offer enhanced extraction efficiency, reduced environmental impact, and preservation of delicate bioactive compounds, significantly improving the quality and consistency of *C. asiatica*-derived products.

Concurrently, progress in analytical chemistry, particularly high-performance liquid chromatography (HPLC), mass spectrometry (MS), and nuclear magnetic resonance (NMR) spectroscopy, has enabled the precise characterization of key phytochemicals, such as asiaticoside, madecassoside, and asiatic acid [7,8]. These tools not only ensure batch-to-batch reproducibility but also serve as foundational pillars for product standardization and regulatory approval.

Despite these advancements, critical gaps persist. Most commercial *C. asiatica* products lack standardization, with variable phytochemical content, questionable bioavailability, and limited clinical evidence supporting specific indications [9]. Moreover, the majority of studies remain preclinical, often using crude extracts or isolated compounds without clear translational pathways. This highlights the urgent need for rigorous clinical trials, pharmacokinetic evaluations, and integrative formulation approaches (e.g., nano-delivery systems) to fully realize the plant’s therapeutic promise.

Beyond pharmacological utility, the integration of *C. asiatica* into nutraceuticals, cosmeceuticals, and functional foods illustrates its versatility. However, commercialization must be balanced with sustainable sourcing, conservation, and equitable benefit-sharing, particularly in regions where *C. asiatica* is cultivated traditionally. Thus, in this review, we critically examine the technological, scientific, and regulatory milestones shaping the modern use of *C. asiatica*. By presenting an integrated overview of its extraction technologies, phytochemical constitution, therapeutic applications, and product development strategies, we aim to provide a valuable resource for researchers, clinicians, product developers, and policymakers navigating the complex terrain of herbal medicine modernization.

### Literature Search Strategy

A rigorous literature search was carried out to ensure comprehensive coverage of unique extraction procedures used for *Centella asiatica*. The authors searched databases such as PubMed, Scopus, Web of Science, ScienceDirect, and Google Scholar using the following keywords: *Centella asiatica*, extraction methods, supercritical fluid extraction, microwave-assisted extraction, ultrasound-assisted extraction, enzyme-assisted extraction, and bioactive compound isolation. The search filters included works published from 2010 to 2025, written in English, with a focus on experimental research articles, review papers, and case studies that explain extraction parameters, yield outcomes, and chemical profiles.

## 2. Global Distribution, Ecological Adaptability, and Cultural Significance

*C. asiatica* is native to the wetlands of Asia and has demonstrated remarkable ecological plasticity, enabling its spread across tropical and subtropical regions worldwide. Naturally occurring in countries such as India, Sri Lanka, China, Indonesia, Madagascar, and South Africa, the species thrives in humid environments, including marshes, riverbanks, paddy fields, and forest undergrowth [10]. It exhibits a strong preference for partial shade and soils rich in organic content, often colonizing loamy or clayey substrates with high moisture retention [11].

**Figure 1:**
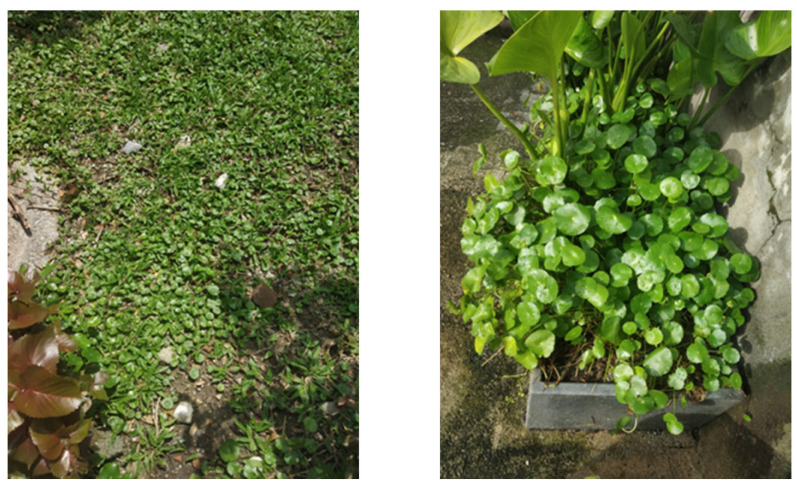
*Centella asiatica*. (**Left**) Wild *Centella asiatica*. (**Right**) Original, photographed at the captive flora.

This wide distribution is attributed to the plant’s efficient clonal reproduction through stolons, as well as its tolerance to varying soil pH, flooding, and moderate salinity (Figure 1). Its success as a cosmopolitan species makes it not only a phytotherapeutic resource but also a valuable subject for ecological and agricultural studies related to climate resilience and sustainable land use [11,12]. In Malaysia, *C. asiatica*, known by various regional names such as “Pegaga” and “Pegaga gajah,” takes center stage in both the country’s ecology and culture. The plant’s distribution in Malaysia is extensive and plays an important role within the nation’s landscapes. It can be found in multiple states, including Penang, Perak, Kedah, Kelantan, and Terengganu [12]. The northern states of Peninsular Malaysia have a rich, abundant growth of this remarkable plant. Beyond its botanical importance, Pegaga holds cultural significance in Malaysia. It has been a part of Malaysian traditional medicine and is highly regarded for its healing properties.

Additionally, Pegaga is celebrated as a culinary ingredient, adding a unique flavor to various traditional dishes and beverages. Its adaptability is further highlighted in natural habitats and human-made landscapes (Figure 1). Pegaga flourishes in lush wetlands, forested areas, and along the riverbanks of the country, and it is also actively cultivated in home gardens and agricultural fields (Figure 1). Finally, Pegaga’s multifaceted benefits shine brightly, with both traditional healers and herbalists recognizing its therapeutic properties and the importance of this botanical wonder and the people of Malaysia [12,13].

## 3. Taxonomy and Botanical Description

**Figure 2:**
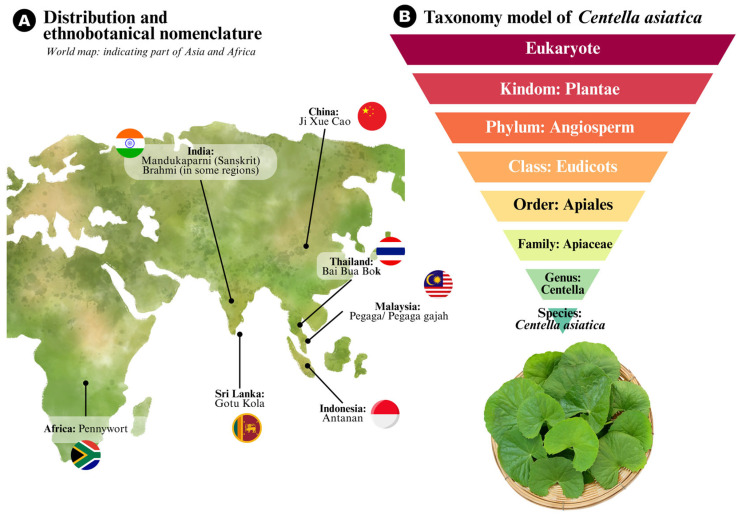
Illustration of (**A**) the global distribution (map) and ethnobotanical nomenclature of *Centella asiatica*, and (**B**) taxonomical model of *Centella asiatica*.

*C. asiatica* belongs to the Apiaceae family (formerly Umbelliferae), a large family of flowering plants that includes numerous aromatic and medicinal species. Its taxonomy classification is as shown in Figure 2. This species was previously grouped under the genus *Hydrocotyle*, which led to significant confusion due to morphological similarities [14]. However, molecular phylogenetic analyses have since confirmed *Centella* as a distinct genus within the Apiaceae family, differentiated by unique floral and leaf characteristics. Notably, *C. asiatica* possesses solitary, axillary inflorescences with small, pink to purple umbellate flowers and kidney-shaped to fan-shaped leaves with crenate or scalloped margins. The leaf morphology, in particular, serves as a diagnostic feature in differentiating this species from closely related taxa and ecological mimics [14].

The genus *Centella* comprises approximately 40 species, most of which are distributed in tropical and subtropical regions. However, *C. asiatica* is the most pharmaceutically and economically significant among them, often used synonymously with “Gotu Kola” in medicinal and commercial contexts. Moreover, *C. asiatica* is known by numerous vernacular names, reflecting its widespread use and cultural integration (see Figure 2). These diverse names underscore its global recognition and ethnomedicinal importance. However, this linguistic diversity also poses challenges for pharmacognostic standardization, reinforcing the need for clear botanical identification in both traditional use and commercial contexts.

Furthermore, *C. asiatica* is diploid with a chromosome number of 2n = 18, whereby it exhibits moderate intraspecific variation, particularly in phytochemical profiles, which can be influenced by geographic origin, genotype, and cultivation practices [15]. Molecular studies using randomly amplified polymorphic DNA (RAPD) [16], amplified fragment length polymorphism (AFLP) [17], and simple sequence repeats (SSR) [18] markers have revealed significant genotypic diversity across regional populations, suggesting the importance of genetic conservation and germplasm standardization for breeding and pharmacological research.

## 4. Conservation Status

*C. asiatica* is currently classified as a species of “Least Concern” by the International Union for Conservation of Nature (IUCN), reflecting its broad global distribution and relatively stable population trends [19]. However, this designation may obscure region-specific vulnerabilities and emerging threats, particularly in areas where the plant is heavily harvested for commercial purposes or faces habitat degradation due to agricultural expansion and urban development.

Although *C. asiatica* is naturally resilient and adaptable to a range of habitats, the increasing demand for its use in pharmaceuticals, cosmeceuticals, and nutraceuticals has placed substantial pressure on wild populations (i.e., ecological pressures) and overexploitation. In countries such as India, Sri Lanka, and Malaysia, unsustainable harvesting from the wild, often without replanting or adherence to conservation guidelines, has led to localized declines in plant density, reduced reproductive viability, and compromised phytochemical quality due to genetic depletion [20,21]. Moreover, habitat loss from deforestation, water pollution, and wetland drainage poses additional threats to native populations. The disruption of riparian and wetland ecosystems, in particular, undermines the plant’s ecological niche, threatening both wild gene pools and local biodiversity.

Additionally, given its economic value and pharmacological promise, the long-term viability of *C. asiatica* depends on transitioning from wild collection to controlled, sustainable cultivation. Good Agricultural and Collection Practices (GACP) as recommended by the World Health Organization (WHO) should be implemented across source regions [22]. These include standardized cultivation protocols, soil and irrigation management, and post-harvest handling to ensure consistent phytochemical yields and environmental stewardship. In parallel, efforts should focus on ex situ conservation, including: (1) Seed banking and tissue culture propagation for preserving genetic diversity; (2) Clonal propagation and micropropagation to maintain elite chemotypes with high bioactive content; and (3) Establishment of conservation plots and botanical gardens as living repositories for long-term access and research.

Moreover, to complement scientific and agronomic solutions, community-based conservation programs should be prioritized. These may involve: (1) Training local harvesters in sustainable collection techniques; (2) Providing incentives for farmers to engage in organic or certified cultivation; and (3) Integrating traditional ecological knowledge with modern agroforestry models to preserve both biodiversity and cultural heritage. Finally, regional and international cooperation is necessary to develop biodiversity access and benefit-sharing frameworks under the Convention on Biological Diversity (CBD) and the Nagoya Protocol, ensuring equitable distribution of commercial and medicinal benefits derived from *C. asiatica* [23,24].

Finally, despite its popularity, comprehensive ecological and genetic studies on *C. asiatica* are limited. Key research gaps include: (1) Population-level assessments of genetic diversity and resilience; (2) Impact of climate change on habitat suitability and phytochemical variation; and (3) Standardized protocols for propagation and domestication across different agroecological zones. Addressing these knowledge gaps will be critical for ensuring the long-term sustainability of *C. asiatica* as a medicinal resource and for preserving its role in traditional medicine systems.

## 5. Phytochemistry of *C. asiatica*

*C. asiatica* exhibits a rich and diverse phytochemical profile, dominated by triterpenoid saponins, flavonoids, phenolic acids, alkaloids, phytosterols, volatile oils, and polyacetylenes [25]. These compounds underpin the plant’s broad pharmacological actions, including wound healing, neuroprotection, anti-inflammatory, antioxidant, anti-aging, and adaptogenic effects. Recent advancements in metabolomics [26], ultra-performance liquid chromatography (UPLC) [27], NMR [28], and MS, e.g., liquid chromatography-MS (LC-MS/MS) and gas chromatography-MS (GC-MS) [29], have expanded the known chemical space of *C. asiatica*, enabling deeper insight into compound bioavailability, pharmacodynamics, and standardization for therapeutic use.

### 5.1. Triterpenoid Saponins: Signature Phytochemicals

Triterpenoid saponins are the hallmark bioactives of *C. asiatica*, mainly falling into two major groups: asiaticosides and madecassosides, derived from their respective aglycones, such as asiatic acid and madecassic acid.

#### 5.1.1. Asiaticoside

**Figure 3:**
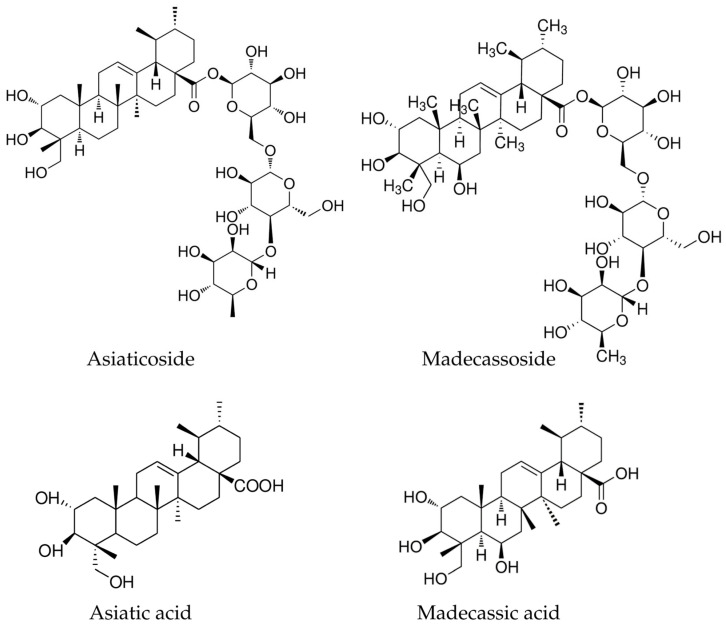
Chemical structures of major bioactive triterpenoid compounds isolated from *Centella asiatica*. The figure depicts the structures of asiaticoside, madecassoside, asiatic acid, and madecassic acid. Asiaticoside and madecassoside are glycosylated forms, while asiatic acid and madecassic acid represent their corresponding aglycones. These compounds are primarily responsible for the plant’s pharmacological activities, including neuroprotective, wound-healing, antioxidant, and anti-inflammatory effects.

Asiaticoside is one of the most well-known triterpenoids and has been the subject of intensive research due to its potential for promoting collagen synthesis and wound healing, and also because of its potent anti-inflammatory effects [30,31]. The chemical structure of asiaticoside is a triterpene glycoside consisting of a triterpenoid aglycone and a glucose molecule (molecular formula of asiaticoside is C_48_H_78_O_19_) (Figure 3). The aglycone part of asiaticoside is asiatic acid, a pentacyclic triterpenoid, and the multiple hydroxyl (OH) groups confer further complexity to the multi-ring structure [32].

Asiaticoside’s capacity to stimulate collagen synthesis by upregulating transforming growth factor beta 1 (TGF-β1) and fibronectin enhances the formation of new blood vessels (angiogenesis) and makes a significant contribution to tissue regeneration. These effects make asiaticoside particularly useful in treating various skin conditions, including wounds, burns, and scars [31], consequently garnering considerable interest in scientific research through its medicinal properties [31]. Moreover, recent studies demonstrate asiaticoside’s role in promoting neurogenesis via brain-derived neurotrophic factor (BDNF) upregulation, suggesting potential for neurodegenerative disease therapies [33].

#### 5.1.2. Madecassoside

Madecassoside is another important triterpenoid found in *C. asiatica*. Madecassoside is a glycoside, specifically a triterpene saponin, consisting of a triterpenoid aglycone, madecassic acid, and a sugar moiety, usually glucose, resulting in the molecular formula C_48_H_78_O_20_; its structure closely mimics that of asiatic acid (Figure 3) [34]. Like asiaticoside, it also plays a significant role in inhibiting nuclear factor kappa B (NF-κB) activation, reduces cytokine-induced inflammation, protects against UV-induced skin aging, and aids in wound healing [35,36]. Moreover, it is also known for its anti-inflammatory properties and ability to promote collagen production, which is crucial for skin repair [3]. Recent studies have also shown that madecassoside-loaded nanoparticles have improved transdermal delivery and stability in dermatological applications [37].

#### 5.1.3. Asiatic Acid and Madecassic Acid

Asiatic acid (C_30_H_48_O_5_) and madecassic acid (C_30_H_48_O_6_) are pentacyclic triterpenoids. They exhibit anti-inflammatory, anticancer, hepatoprotective, and neuroprotective properties via modulation of mitogen-activated protein kinases (MAPK), nuclear factor erythroid 2-related factor 2 (Nrf2), and phosphatidylinositol 3-kinase/protein kinase B (PI3K/Akt) signaling pathways [38]. A recent study indicated that asiatic acid derivatives have been investigated as scaffolds for anticancer drug development due to their cytotoxicity against glioblastoma and melanoma cells [39]. Likewise, madecassoside acid exhibits anti-inflammatory and antioxidant effects, which can contribute to the plant’s overall wound-healing properties [40].

Additionally, though not as extensively studied, preliminary phytochemical assessments described brahmoside as one of multiple pentacyclic triterpene glycosides in *C. asiatica*, particularly in chemotypes from India and Southeast Asia [25]. Additionally, early pharmacological studies suggest that brahmoside may have potential anxiolytic and adaptogenic effects [41]. However, comprehensive mechanistic and clinical data remain limited, warranting further investigation into its therapeutic relevance and standardization across extract formulations.

In summary, triterpenoids, in general, are known for their diverse pharmacological activities, including anti-inflammatory, antioxidant, and wound-healing effects. In the case of *C. asiatica*, these triterpenoids are believed to work together to promote skin health, tissue repair, and overall well-being. Studies indicate that whole-extract formulations containing multiple triterpenoids show superior efficacy compared to isolated compounds, suggesting synergistic modulation of inflammatory and oxidative pathways [42]. While the mechanisms of action are not fully understood, ongoing research sheds light on the specific pathways through which these triterpenoids exert their beneficial effects.

### 5.2. Flavonoids and Other Polyphenols

*C. asiatica* is particularly rich in flavonoids, a class of compounds that play a central role in its antioxidant, anti-inflammatory, neuroprotective, and vasoprotective activities [7]. Among these, quercetin is one of the most prominent, known for its ability to scavenge reactive oxygen species (ROS) and modulate key inflammatory mediators, such as cyclooxygenase-2 (COX-2) and tumor necrosis factor alpha (TNF-α) [43,44]. In addition to its antioxidative effects, quercetin has demonstrated neuroprotective activity in experimental models of Alzheimer’s disease, indicating its relevance in cognitive and age-related disorders [45].

Kaempferol, another major flavonoid present in *C. asiatica*, exhibits potent anti-inflammatory properties, primarily by inhibiting the NF-κB signaling pathway and activating Nrf2-mediated antioxidant responses [46]. Rutin, widely recognized for its ability to strengthen capillary integrity, also contributes to the herb’s antioxidative and UV-protective effects, making it particularly valuable in dermatological and vascular applications [47,48]. Other flavonoids such as apigenin, luteolin, and hesperidin are also found in the plant and contribute to its broad therapeutic potential by offering additional anti-aging, anxiolytic, and antimicrobial properties [49].

Recent studies have provided novel insights into the collective role of flavonoid-rich *C. asiatica* extracts in neurological health. These extracts have been shown to reduce β-amyloid aggregation and enhance synaptic plasticity, suggesting a promising role in the management of neurodegenerative conditions and cognitive decline [50,51]. The diverse structural profiles and synergistic bioactivity of these flavonoids enhance the therapeutic value of *C. asiatica* and continue to attract interest for their potential in evidence-based phytomedicine. As research progresses, the full spectrum of flavonoid compounds in *C. asiatica* is being elucidated, deepening our understanding of their mechanisms and supporting the standardization of flavonoid-rich extracts for targeted clinical applications.

### 5.3. Alkaloids

Although alkaloids are present in *C. asiatica* in relatively low abundance compared to triterpenoids and flavonoids, they constitute an important component of the plant’s secondary metabolite spectrum. Several unique alkaloids have been identified, including hydrocotyline and thankuniside [52]. While their concentrations are typically minor, these compounds are increasingly recognized for their potential pharmacological relevance, particularly in the context of neuroprotective and nootropic effects.

Emerging evidence suggests that some of these alkaloids may exhibit cholinesterase inhibitory activity, positioning them as promising candidates for further exploration in the management of neurodegenerative disorders such as Alzheimer’s disease [53,54]. Their role in modulating central nervous system function, possibly through neurotransmitter regulation or neuroinflammation pathways, remains an active area of investigation. Despite this potential, the biological actions and mechanisms of alkaloids in *C. asiatica* are still poorly defined compared to the extensively studied triterpenoids and flavonoids. As a result, current therapeutic applications and product development efforts continue to emphasize the latter compound classes. Nonetheless, the inclusion of alkaloids adds to the plant’s phytochemical complexity and may contribute subtle but synergistic effects to its overall therapeutic profile.

Continued pharmacological and mechanistic studies are warranted to clarify the significance of these lesser-studied constituents and determine whether they offer additive or unique bioactivities that could complement the known effects of other major compounds in *C. asiatica*.

### 5.4. Essential Oils and Volatile Compounds

The essential oil fraction of *C. asiatica*, though present in relatively small amounts (typically ranging from 0.1% to 0.2% *w*/*w*), contributes meaningfully to the plant’s overall therapeutic profile. These oils comprise a variety of monoterpenes and sesquiterpenes, such as caryophyllene, α-humulene, and β-pinene [7,55]. While these constituents are not considered the primary pharmacologically active agents of the plant, their presence introduces additional dimensions to its bioactivity, particularly in relation to antimicrobial, anxiolytic, and dermal applications [55].

The aromatic compounds within the essential oil component are known to exert mild calming, stress-relieving, and skin-soothing effects, which align with *C. asiatica*’s traditional use in holistic medicine systems. The potential anxiolytic and mood-stabilizing properties of these volatiles, although less rigorously studied than those of the plant’s triterpenoids and flavonoids, are consistent with reports from aromatherapy and traditional formulations [54].

Although essential oils are acknowledged as permeation enhancers in transdermal systems, aiding the absorption of lipophilic substances like triterpenoids, the precise function of *C. asiatica* essential oil in this context is little defined. Due to the very low concentration of essential oil in most *Centella* preparations, additional research is necessary to confirm its role in improving cutaneous penetration [56]. Such innovations support the integrative use of essential oils not merely for their intrinsic effects but also for their functional contributions to compound delivery.

It is important to note that the composition and yield of essential oils in *C. asiatica* can vary significantly depending on multiple factors, including geographic origin, soil composition, harvesting time, and the specific plant part used (leaves, stems, or roots) [52]. Among them, the leaves were discovered to have the largest concentration of essential oils, particularly monoterpenes and sesquiterpenes, which contribute to their biological activity and permeation-enhancing capacity [52]. Despite their lower abundance relative to major phytochemical groups, these volatile oils enhance the organoleptic properties of *C. asiatica*-based products and may contribute synergistically to their therapeutic experience [52]. However, in the broader context of the plant’s medicinal potential, essential oils are considered secondary in importance to the dominant bioactive categories of triterpenoids and flavonoids.

### 5.5. Phytosterols

Phytosterols are naturally occurring, plant-derived sterols structurally similar to cholesterol, and they play a noteworthy role in the pharmacological landscape of *C. asiatica*. Although often overshadowed by the plant’s more prominent triterpenoids, flavonoids, and alkaloids, phytosterols represent an important class of bioactive compounds that contribute to its broader therapeutic potential [7,52].

The major phytosterols identified in *C. asiatica* include β-sitosterol, campesterol, and stigmasterol, which are mostly stored in the leaves, with moderate levels in the stems and roots [52]. These compounds are widely recognized for their ability to modulate lipid metabolism, primarily by competing with dietary cholesterol for absorption in the gastrointestinal tract [57]. This competitive mechanism effectively lowers circulating cholesterol levels, thereby offering protective effects against cardiovascular diseases. In addition to their hypocholesterolemic activity, phytosterols also exhibit anti-inflammatory and antioxidant properties, which further support their utility in managing metabolic syndrome and related inflammatory conditions [58].

Recent pharmacological investigations have also highlighted the immunomodulatory potential of these compounds, suggesting that phytosterols may influence immune cell regulation and cytokine expression, though more mechanistic studies are warranted in the context of *C. asiatica*-derived formulations [59,60]. While not traditionally the central focus in discussions of the plant’s medicinal value, phytosterols complement the actions of other bioactives by contributing to systemic effects on cardiovascular, metabolic, and immune health. Their inclusion in the phytochemical profile of *C. asiatica* enhances the plant’s versatility and substantiates its use in integrative medicine, particularly in formulations targeting lipid regulation and chronic inflammation.

### 5.6. Polyacetylenes

Polyacetylenes, a lesser-known class of bioactive compounds, form part of the diverse phytochemical repertoire of *C. asiatica*. These compounds, though present in lower abundance compared to triterpenoids and flavonoids, are primarily localized in the roots, with moderate presence in the leaves, thereby contributing meaningfully to the plant’s therapeutic complexity. Among the identified polyacetylenes in *C. asiatica* are centellin, isocentellin, and related acetylenic derivatives [7,52]. These molecules possess structurally reactive triple bonds, which confer distinct bioactivities that are gaining recognition in both phytochemical and pharmacological research.

Recent studies have demonstrated that polyacetylenes, such as cadiyenol and cadiyenone, can promote fibroblast migration, stimulate angiogenesis, and enhance skin regeneration properties, which align closely with *C. asiatica*’s established role in wound healing and dermatological formulations [61,62]. Their presence may potentiate the wound-repair cascade, possibly through modulation of extracellular matrix remodeling and cellular signaling involved in tissue repair. Beyond these dermatological effects, a novel perspective has emerged suggesting that polyacetylenes may function as endogenous signaling molecules in plants, particularly in response to environmental stressors. This signaling role, crucial for plant defense and adaptation, may be pharmacologically relevant in humans as well, potentially contributing to *C. asiatica*’s adaptogenic properties by modulating cellular stress responses [63,64].

Although research into the mechanisms and clinical relevance of polyacetylenes remains in its early stages, their bioactivity and structural uniqueness underscore the importance of considering these compounds in future investigations. A deeper understanding of their role could expand the therapeutic applications of *C. asiatica* and provide new targets for phytopharmaceutical development.

### 5.7. Biosynthesis and Biotechnological Perspectives

The biosynthesis of triterpenoids in *C. asiatica* predominantly occurs via the mevalonate pathway, a key metabolic route in plants responsible for the generation of isoprenoid compounds. Central to this pathway are enzymes such as β-amyrin synthase, which catalyzes the formation of the triterpenoid skeleton, and cytochrome P450 oxidases, which introduce functional modifications essential for bioactivity [65]. Recent advances in transcriptomic and metabolomic analyses have identified specific gene clusters that regulate the biosynthetic machinery of major bioactive compounds, including asiaticoside and madecassoside [65]. These findings have opened new avenues for metabolic engineering aimed at enhancing the yield and consistency of targeted phytochemicals.

In the realm of applied biotechnology, significant progress has been made in developing plant tissue culture systems, such as cell suspension cultures and hairy root cultures, which allow for the scalable and controlled production of triterpenoids under laboratory and industrial conditions [66]. These systems offer advantages in yield optimization, compound consistency, and environmental sustainability. Moreover, cutting-edge molecular approaches, including CRISPR-Cas9 genome editing and transgenic manipulation, are currently being explored to upregulate key biosynthetic genes and improve the metabolic flux toward high-value secondary metabolites [67]. Such innovations represent a promising frontier in ensuring a sustainable and standardized supply of *C. asiatica*-derived compounds for therapeutic use.

### 5.8. Chemotypic Variation and Standardization Challenges

Despite its growing popularity and wide distribution, *C. asiatica* exhibits substantial variability in its phytochemical composition, which poses significant challenges for product standardization and therapeutic reproducibility. The concentration and profile of bioactive compounds can fluctuate markedly based on several interrelated factors, including the plant’s geographic origin, the quality and type of soil in which it is cultivated, seasonal and environmental conditions during growth, and the timing of harvest [11,52].

Additionally, the specific plant part used, whether leaf, root, or stem, has a measurable impact on phytochemical distribution, as each tissue type can express different concentrations of triterpenoids, flavonoids, and other secondary metabolites [68]. The extraction technique applied further influences the final composition, with differences in solvent polarity, temperature, and extraction duration contributing to variations in yield and compound integrity [69].

Such chemotypic diversity complicates the development of uniform formulations and undermines the reproducibility of clinical outcomes. Therefore, it is imperative to implement robust quality control strategies, including the use of chemotypic markers, validated HPLC fingerprinting protocols, and adherence to pharmacopoeia monographs [70]. These tools are essential for ensuring batch-to-batch consistency and for supporting regulatory compliance in the development of standardized *C. asiatica*-based therapeutics.

**Figure 4:**
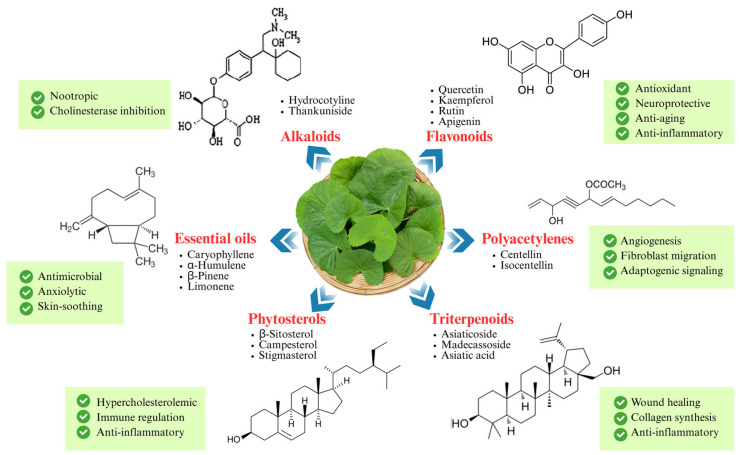
Major phytochemical classes in *Centella asiatica* and their associated bioactivities (in green box).

In summary, the phytochemistry of *C. asiatica* is both complex and dynamic, offering a chemically rich foundation for a wide range of pharmacological effects. A summary of major phytochemical classes in *C. asiatica* and their associated bioactivities is illustrated in Figure 4. Ongoing innovations in analytical chemistry, biotechnology, and systems biology are not only deepening our understanding of these compounds but also paving the way for their optimized application in evidence-based herbal medicine. By integrating compound synergy, biosynthetic pathways, and novel formulation strategies, researchers can unlock the full therapeutic potential of this versatile plant.

## 6. Advanced Extraction Techniques

In the pursuit of unlocking the therapeutic potential of *C. asiatica*, advanced extraction techniques have become instrumental in optimizing the yield of bioactive compounds. Numerous studies have empirically validated the effectiveness of improved extraction techniques in improving the recovery of triterpenoids and other bioactive compounds from *C. asiatica*. Techniques such as SFE, MAE, UAE, enzyme-assisted extraction (EAE), and pressurized liquid extraction (PLE) stand at the forefront of this innovative frontier [5]. Each method uniquely exploits physical and chemical principles to extract valuable compounds from plant material, showcasing benefits such as enhanced selectivity, reduced extraction times, and environmentally friendly approaches. However, these techniques are not without their challenges, including equipment costs, operational complexities, and the need for meticulous optimization. This exploration of advanced extraction methods for *C. asiatica* exhibits a dynamic intersection between traditional herbal understanding and modern technological prowess, offering a pathway to unlock the full therapeutic potential of this medicinal plant [69].

### 6.1. Supercritical Fluid Extraction (SFE)

SFE has emerged as a powerful and environmentally friendly technique for extracting bioactive compounds from *C. asiatica*. The principles underlying SFE involve using supercritical fluids, commonly carbon dioxide (CO_2_), at conditions surpassing critical temperature and pressure [71]. In the methodology employed for *C. asiatica*, selecting optimal CO_2_ pressure and temperature is crucial and greatly influences the extraction efficiency. The introduction of co-solvents, as required, enhances the solubility of specific bioactive compounds. Extraction time is optimized to achieve maximum yield while preserving the plant’s more labile compounds.

The advantages of SFE in *C. asiatica* extraction are notable. This method allows for the selective extraction of target compounds as more refined and concentrated extracts. Moreover, supercritical CO_2_ minimizes thermal degradation, making it particularly useful for preserving the heat-sensitive bioactivity of the extract [72]. In addition, the non-polluting nature of CO_2_, being non-toxic and easily removable, aligns with the growing emphasis on sustainable and green extraction processes, further establishing SFE as a leading technique in the quest for high-quality *C. asiatica* extracts [72].

### 6.2. Microwave-Assisted Extraction (MAE)

MAE is a cutting-edge method for extracting bioactive compounds. The underlying principle involves using microwave radiation to induce localized heating, expediting the extraction process. In this method, optimizing microwave power and extraction time is paramount to achieve an efficient extraction while avoiding the thermal degradation of sensitive compounds [73]. The careful selection of suitable solvents enhances the absorption of microwaves, contributing to the overall efficacy of the extraction.

Noteworthy is the rapidity of the MAE process, which significantly reduces extraction time compared to traditional methods, making it particularly advantageous for *C. asiatica*, where the preservation of labile bioactive compounds is crucial. The advantages extend to a reduction in solvent consumption, aligning with the broader sustainability goals in extraction processes. Moreover, the application of MAE in conjunction with LC-MS/MS facilitated the effective measurement of certain triterpenes and caffeoylquinic acids while minimizing heat degradation [5]. As an innovative and efficient technique, MAE demonstrates promise in unlocking the full therapeutic potential of *C. asiatica*, offering a pathway to more robust and time-efficient herbal extract production [74].

Furthermore, green extraction technologies, such as SFE and MAE, exhibit enhanced phytochemical retention and environmental sustainability, particularly when ethanol is employed as a co-solvent [6]. Apart from that, SFE was identified to improve selectivity and yield while eliminating solvent residues, establishing it as a favored method for high-value extract formulations [18]. In a separate investigation, MAE markedly enhanced the recovery of asiatic acid while maintaining antioxidant activity [69].

### 6.3. Ultrasound-Assisted Extraction (UAE)

UAE has emerged as a highly effective technique for extracting bioactive compounds from *C. asiatica*, which leverages the principles of ultrasonic waves to induce cavitation and enhance the release of valuable compounds from plant tissues. The foundational principle involves the creation of cavitation bubbles through ultrasonic waves, disrupting cell walls and facilitating the extraction of bioactive constituents. In the methodology designed for *C. asiatica*, optimizing ultrasound frequency and intensity are crucial in influencing the efficiency of the extraction process [75]. The careful selection of appropriate solvents further contributes to the UAE’s overall success by enhancing target compounds’ solubility.

The UAE, employing 70% ethanol, has demonstrated considerable enhancement in the yield of asiaticoside and madecassoside, while concurrently decreasing solvent usage and processing duration [5]. Additionally, controlling extraction time and temperature is paramount to preventing thermal degradation and preserving labile bioactive compounds. UAE offers several advantages in the context of *C. asiatica* extraction. The induced cavitation promotes increased mass transfer, producing higher extraction rates and yielding extracts with enhanced bioactive content. Furthermore, the UAE demonstrates a remarkable ability to preserve thermolabile compounds, ensuring the retention of the plant’s intricate biochemical profile. Notably, reducing solvent consumption aligns with sustainability goals, making the UAE a promising and eco-friendly method for obtaining high-quality *C. asiatica* extracts [76].

### 6.4. Enzyme-Assisted Extraction (EAE)

EAE emerges as an innovative strategy for extracting bioactive compounds from *C. asiatica*, capitalizing on the fundamental principle that enzymes facilitate the breakdown of cell walls and membranes, promoting the release of intracellular compounds [77,78]. In the methodology tailored for *C. asiatica*, selecting enzymes is critical, emphasizing specificity and efficiency to ensure targeted extraction. The optimization of enzyme concentration and incubation plays a pivotal role in enhancing the efficiency of the extraction process, allowing for the selective release of bioactive constituents. Post-extraction, careful inactivation of enzymes is implemented to prevent any potential degradation of the extracted compounds.

The advantages of EAE in the context of *C. asiatica* are noteworthy. This method allows for the selective extraction of specific compounds, providing a more refined and targeted approach to obtaining desired active components [79]. The use of mild extraction conditions ensures the preservation of the plant’s inherent bioactivity, which is particularly crucial for delicate compounds in *C. asiatica*. Moreover, the reduced environmental impact, stemming from the specificity of the enzyme action and the minimized use of harsh solvents, aligns with sustainable extraction practices, making EAE a promising avenue for obtaining high-quality *C. asiatica* extracts with a minimal ecological footprint [77,78].

### 6.5. Pressurized Liquid Extraction (PLE)

PLE is a robust method for extracting bioactive compounds from *C. asiatica*, leveraging high pressure and temperature to enhance solvent penetration into the plant material. The foundational principles involve creating an environment of elevated pressure and temperature, facilitating the efficient extraction of valuable compounds [79]. In the methodological framework developed for *C. asiatica*, optimizing key parameters, including stress, temperature, and solvent type, is crucial for achieving optimal extraction efficiency. Careful control of extraction time is also essential to prevent thermal degradation of sensitive compounds.

The advantages of PLE in the context of *C. asiatica* extraction include the efficient extraction of various combinations, ensuring a comprehensive plant bioactive profile representation [80]. Notably, PLE offers a significant reduction in extraction time compared to traditional methods, streamlining the overall extraction process without compromising the quality of the obtained extracts. Furthermore, PLE exhibits high reproducibility, contributing to the reliability and consistency of the extraction process. The integration of PLE into *C. asiatica* extraction protocols present a promising avenue for obtaining high-quality extracts with efficiency, reduced processing time, and enhanced reproducibility [79,80].

Extraction of bioactive compounds from *C. asiatica* using advanced techniques offers distinct advantages and limitations (Table 1). SFE utilizes supercritical fluids, typically CO_2_, enabling selective extraction with minimal thermal degradation, making it an eco-friendly approach. MAE is recognized for its rapid processing time, significantly reducing extraction duration; however, it requires careful temperature control to prevent overheating and degradation of sensitive compounds. UAE employs ultrasonic waves to enhance mass transfer and effectively preserve thermolabile constituents. EAE provides specificity through the enzymatic breakdown of plant cell walls, facilitating targeted release of bioactive compounds, though it often necessitates subsequent enzyme inactivation steps. PLE, conducted under high pressure and temperature, efficiently extracts a broad spectrum of compounds in reduced time but requires optimization to avoid thermal damage.

While these techniques improve extraction efficiency and bioactive compound preservation, practical considerations, including equipment costs, operational complexity, and challenges in scale-up, must be carefully balanced. Selecting the most appropriate method should therefore align with specific research goals and practical constraints. From a sustainability perspective, green extraction principles prioritize minimal solvent use, low energy consumption, and reduced environmental impact [81]. SFE and EAE stand out for their sustainability profiles, utilizing non-toxic, food-grade solvents such as CO_2_ and water and operating under mild, energy-efficient conditions. UAE also supports green chemistry goals due to its low energy demand and short processing times. Solvent sustainability is critical; CO_2_ (SFE), water (EAE/UAE), and ethanol (MAE) are biodegradable and approved by regulatory agencies, facilitating scalable and compliant production processes. Avoiding chlorinated solvents aligns *C. asiatica* extraction with WHO and EMA phytopharmaceutical standards [5,81].

Regarding industrial scalability, MAE, UAE, and PLE are highly suitable for large-scale production because of their rapid processing, reproducibility, and compatibility with automation. Although SFE requires higher capital investment, it offers excellent selectivity and produces solvent-free extracts, making it ideal for high-value formulations. EAE, while less scalable as a standalone method, is valuable in combination with other techniques.

Moreover, hybrid extraction strategies, such as MAE–EAE (combining rapid thermal diffusion with enzyme specificity) and SFE–PLE (targeting diverse phytochemical phases), demonstrate synergistic benefits. These integrated approaches maximize yield, preserve compound integrity, and exemplify the fusion of efficiency, sustainability, and phytochemical fidelity in next-generation *C. asiatica* extraction methods [5,70,75].

## 7. Therapeutic Applications

### 7.1. Molecular Mechanisms Underpinning Therapeutic Actions

The therapeutic versatility of *C. asiatica* is largely attributed to its ability to modulate several interconnected molecular pathways that regulate inflammation, oxidative stress, cellular repair, and apoptosis. These mechanisms are mediated predominantly by their key bioactive constituents, including triterpenoid saponins such as asiaticoside and madecassoside, as well as asiatic acid. One of the central pathways affected by *C. asiatica* is the NF-κB signaling pathway [82]. This transcription factor plays a pivotal role in orchestrating the inflammatory response. Bioactive compounds from *C. asiatica* inhibit the activation and nuclear translocation of NF-κB, resulting in the downregulation of pro-inflammatory cytokines such as interleukin-1β (IL-1β), IL-6, and TNF-α, as well as the suppression of inflammatory enzymes including COX-2 and inducible nitric oxide synthase (iNOS) [83]. This mechanism underlies the plant’s well-documented anti-inflammatory effects and has been validated in both in vitro and in vivo models of chronic inflammation [84,85].

In parallel, *C. asiatica* activates the Nrf2 pathway, which serves as a master regulator of cellular antioxidant defense. Through Nrf2 activation, the plant upregulates the expression of key antioxidant enzymes such as superoxide dismutase (SOD), catalase (CAT), and heme oxygenase-1 (HO-1), thereby enhancing the cell’s ability to neutralize ROS and resist oxidative stress [86]. This antioxidant effect plays a crucial role in neuroprotection, skin regeneration, and hepatoprotection [87]. The MAPK pathway is also influenced by *C. asiatica*, particularly through the inhibition of extracellular signal-regulated kinases (ERK1/2) and c-Jun N-terminal kinases (JNK) [85,88]. These MAPKs are critical mediators of cellular stress responses, inflammation, and apoptosis. By modulating these kinases, *C. asiatica* contributes to reduced inflammation, enhanced wound healing, and protection against neural injury.

Moreover, asiatic acid, a major triterpenoid aglycone, has been shown to downregulate the phosphatidylinositol 3-kinase (PI3K)/Akt/mammalian target of rapamycin (mTOR) pathway [89]. This signaling axis is often upregulated in cancer cells, promoting survival and proliferation. Suppression of this pathway by asiatic acid induces apoptosis in various cancer cell lines, suggesting a potential anticancer mechanism that warrants further investigation [88,90].

**Figure 5:**
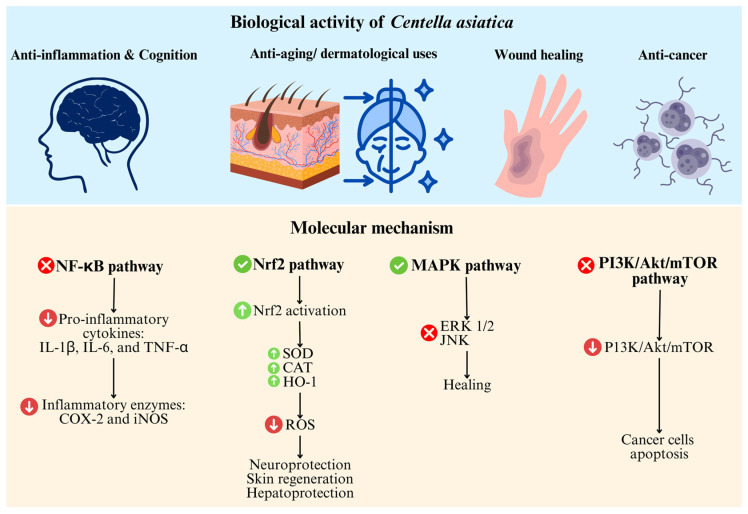
Therapeutic applications and molecular mechanisms of *C. asiatica*. This schematic illustrates the primary therapeutic effects of *C. asiatica*: Anti-inflammatory and cognitive enhancement, anti-aging/dermatological benefits, wound healing, and anticancer activity, linked to modulation of key molecular pathways. *C. asiatica* downregulates the NF-κB pathway to suppress pro-inflammatory cytokines (IL-1β, IL-6, TNF-α) and enzymes (COX-2, iNOS); activates the Nrf2 pathway, enhancing antioxidant enzyme expression (SOD, CAT, HO-1) and reducing ROS; inhibits MAPK (ERK1/2, JNK), promoting wound healing; and suppresses the PI3K/Akt/mTOR axis, inducing apoptosis in cancer cells. NF-κB: nuclear factor kappa-light-chain-enhancer of activated B cells; IL: interleukin; TNF-α: tumor necrosis factor alpha; COX-2: cyclooxygenase-2; iNOS: inducible nitric oxide synthase; Nrf2: nuclear factor erythroid 2–related factor 2; SOD: superoxide dismutase; CAT: catalase; HO-1: heme oxygenase-1; ROS: reactive oxygen species; MAPK: mitogen-activated protein kinase; ERK: extracellular signal-regulated kinase; JNK: c-Jun N-terminal kinase; PI3K/Akt/mTOR: phosphatidylinositol 3-kinase/protein kinase B/mammalian target of rapamycin.

Together, these molecular interactions form the mechanistic basis for *C. asiatica’s* multifaceted pharmacological actions. The integration of anti-inflammatory, antioxidant, regenerative, and anti-proliferative effects makes this botanical an attractive candidate for managing a broad range of conditions, from chronic inflammation and neurodegenerative disorders to wound healing and skin aging (Figure 5).

### 7.2. Clinical Evidence and Meta-Analyses

A growing body of clinical and preclinical research supports the therapeutic potential of *C. asiatica* across a range of health domains, particularly in cognitive function, wound healing, and dermatology. Despite promising outcomes, variability in extract standardization, dosing, and study design continues to limit the generalizability of findings.

In the context of cognitive health and mood regulation, a recent systematic review and meta-analysis reported that supplementation with *C. asiatica* extracts may lead to modest but statistically significant improvements in cognitive performance and anxiety-related symptoms in human subjects [91]. These outcomes are thought to arise from the plant’s ability to enhance neuroplasticity and upregulate BDNF, particularly in models of neurodegeneration and age-associated cognitive decline [33,92]. However, considerable heterogeneity across clinical trials, including differences in extract formulations, dosages (ranging from 300 to 750 mg/day), and cognitive endpoints, poses challenges in drawing definitive conclusions and highlights the urgent need for standardized, high-quality randomized controlled trials [1,93].

In dermatological applications, *C. asiatica* has been extensively investigated for its role in enhancing wound healing and treating skin conditions [94]. Clinical studies have shown that topical application of formulations enriched with madecassoside or whole-plant extracts accelerates wound closure, particularly in diabetic ulcer models [95]. This therapeutic effect is attributed to the stimulation of collagen type I synthesis, fibroblast proliferation, and angiogenic factor upregulation, including fibroblast growth factor (FGF) and vascular endothelial growth factor (VEGF) [94]. These processes collectively support tissue regeneration and epidermal remodeling. Additionally, the plant’s antioxidant and anti-photoaging properties contribute to its widespread incorporation in cosmeceuticals, aimed at mitigating UV-induced dermal damage and maintaining skin integrity [96].

A separate line of clinical evidence supports the use of *C. asiatica* in the management of acne vulgaris. Topical gels and creams containing *C. asiatica* extract have demonstrated efficacy in reducing acne lesion counts, an effect believed to stem from its anti-inflammatory activity, antimicrobial action against skin flora, and promotion of epidermal barrier repair [97]. A systematic review confirmed these findings; however, variability in formulation and treatment duration underscores the need for unified clinical protocols [98].

Overall, while the current literature affirms the broad therapeutic relevance of *C. asiatica*, particularly in cognitive and skin health, inconsistencies in extract characterization and dosage regimens continue to limit clinical translation. Future research must focus on well-designed, large-scale trials using standardized, chemically characterized extracts to validate the reproducibility and efficacy of *C. asiatica*-based interventions across therapeutic categories.

### 7.3. Pharmacokinetics and Dosage Considerations

The pharmacokinetic properties of *C. asiatica*’s active constituents, particularly triterpenoids such as asiaticoside, madecassoside, and their corresponding aglycones, are characterized by low oral bioavailability. This limitation is primarily attributed to poor gastrointestinal absorption, extensive first-pass metabolism, and limited permeability across biological membranes. Studies have shown that these compounds undergo rapid metabolism in the liver and intestines, resulting in reduced systemic concentrations following oral administration [99]. Consequently, topical formulations are often preferred for dermatological applications, as they enable localized delivery of active ingredients directly to target tissues while minimizing systemic exposure and metabolic degradation.

Preclinical studies offer substantial evidence for the conventional renoprotective uses of *C. asiatica*. In a rat model of nephrotoxicity induced by AlCl_3_ and D-galactose, oral administration of *C. asiatica* extract (100–300 mg/kg/day for 70 days) significantly decreased serum creatinine and malondialdehyde levels, restored serum albumin, enhanced antioxidant enzyme activities (SOD and catalase), and ameliorated renal histoarchitecture by reversing tubular and glomerular damage [100]. In support of these findings, an additional investigation utilizing ethanolic leaf extract of *C. asiatica* (200–400 mg/kg/day for 8 days) in a gentamicin-induced nephrotoxicity model exhibited significant enhancements in serum urea, creatinine, and blood urea nitrogen (BUN), as well as normalized urine parameters. Histological assessment verified the preservation of kidney tissue, whereas oxidative stress markers were reduced and antioxidant enzyme levels were elevated [101]. Collectively, these investigations validate the nephroprotective attributes of *C. asiatica* and underscore its potential in addressing drug- and toxin-induced renal injury through processes that encompass oxidative stress control, restoration of enzymatic defenses, and histological repair.

Besides, the hepatoprotective efficacy of *C. asiatica* has been progressively corroborated by in vivo models of chemically induced hepatic damage. A study utilizing AlCl_3_ and D-galactose to induce hepatotoxicity in rats demonstrated that oral administration of *C. asiatica* extract (100–300 mg/kg/day) significantly restored liver enzyme levels (ALT, AST), diminished oxidative stress markers (MDA), augmented antioxidant enzymes (SOD, catalase), and improved hepatocyte architecture indicating protective effects via modulation of oxidative damage and inflammation [102]. In a dimethylnitrosamine-induced hepatic fibrosis model, *C. asiatica* (200 mg/kg/day) was observed to decrease serum ALT, AST, and total bilirubin levels, while histological investigation validated the reduction of fibrosis and inhibition of fibrogenic markers, including TGF-β1 and collagen I [103].

A 2021 comparative investigation further corroborated these findings by evaluating the hepatoprotective activity of *C. asiatica* ethanol extract (CA-HE50) against acute liver injury induced by lipopolysaccharide (LPS) and D-galactosamine in C57BL/6 mice. CA-HE50 therapy resulted in substantial decreases in liver enzyme levels, proinflammatory cytokines (IL-6, TNF-α), and hepatic necrosis. The extract altered inflammatory signaling by downregulating the NF-κB and MAPK pathways and upregulating antioxidant gene expression, including HO-1 and Nrf2 [104]. These investigations collectively illustrate that *C. asiatica* provides hepatoprotective effects via many mechanisms, including augmentation of antioxidants, suppression of inflammation, and antifibrotic activity, underscoring its therapeutic potential in acute and chronic liver injury.

Furthermore, the gastroprotective properties of *C. asiatica* have been confirmed through various experimental models. In a model of ethanol-induced gastric lesions, *C. asiatica* extract (200–400 mg/kg) significantly decreased mucosal damage and lesion index in a dose-dependent manner. The protective effect was linked to elevated prostaglandin E_2_ levels, enhanced mucosal blood flow, and preservation of epithelial integrity [105]. In an indomethacin-induced gastric injury model, *C. asiatica* leaf extract mitigated gastric mucosal damage by modulating inflammatory cytokines (TNF-α, IL-1β), suppressing neutrophil infiltration and oxidative stress, and preserving gastric tight junction proteins [106].

Finally, clinical dosage recommendations for *C. asiatica* vary according to the therapeutic indication and formulation. For cognitive enhancement and mood support, oral administration of standardized extracts in the range of 300 to 750 mg per day has been most frequently employed in clinical studies [1,107]. In wound healing protocols, topical creams containing 1% *C. asiatica* extract are commonly applied twice daily to promote tissue repair and reduce scarring [108]. In acne management, formulations such as gels with extract concentrations between 0.5% and 1% are typically used once or twice daily, demonstrating favorable outcomes in lesion reduction and skin regeneration [109]. Despite these applications, the lack of harmonized dosing guidelines based on pharmacokinetic and pharmacodynamic data underscores the need for further clinical pharmacology research. Table 2 summarizes the dosage and pharmacokinetics of *C. asiatica* extracts and key bioactives.

### 7.4. Limitations and Controversies

Despite its extensive ethnomedicinal use and emerging clinical relevance, the therapeutic application of *C. asiatica* presents several challenges. As discussed, one of the most critical limitations is the inconsistency in extract standardization. Variability in plant sourcing, cultivation conditions, extraction methods, and phytochemical composition has led to significant discrepancies in the concentration and bioavailability of key bioactives across commercial and research-grade preparations. This inconsistency hampers reproducibility in clinical outcomes and complicates regulatory approval processes for herbal products.

Another area of ongoing debate involves the therapeutic efficacy of crude versus purified extracts. Whole-plant preparations may offer the advantage of phytochemical synergy, where multiple constituents interact to produce enhanced or complementary effects [122,123]. However, purified compounds allow for greater pharmacological precision, better dosing control, and more targeted therapeutic outcomes. Determining the optimal approach often depends on the clinical context, desired mechanism of action, and formulation constraints. Further comparative studies are warranted to elucidate whether whole extracts or isolated actives yield superior outcomes in specific indications.

Safety concerns, though relatively rare, also merit consideration. While *C. asiatica* is generally regarded as safe for short-term use, there have been reports of hepatotoxicity associated with prolonged or high-dose ingestion, particularly in susceptible individuals [124]. As a precaution, monitoring of liver function is advisable in patients undergoing extended treatment, especially when oral formulations are used at therapeutic doses over prolonged periods. Moreover, allergic reactions and dermatologic sensitivity have been occasionally reported with topical use, highlighting the importance of individualized risk assessment [125]. In sum, while *C. asiatica* holds considerable therapeutic promise, addressing these limitations through rigorous standardization, pharmacovigilance, and clinical validation will be essential for its broader acceptance in evidence-based medicine.

### 7.5. Safety and Drug-Herb Interactions

While *C. asiatica* is widely used in traditional medicine and generally considered safe when used appropriately, concerns have emerged around its safety profile, especially with prolonged or high-dose use. The most commonly reported adverse effects include mild gastrointestinal discomfort, headache, and skin irritation in topical applications [125,126]. However, isolated reports of hepatotoxicity have raised caution, particularly with extended oral use [124]. These rare but significant events appear to be dose-dependent and may be exacerbated by pre-existing hepatic impairment, underscoring the importance of liver function monitoring during long-term treatment.

In terms of drug-herb interactions, *C. asiatica*’s pharmacological actions suggest potential synergy or interference with conventional medications. Its anti-inflammatory properties, mediated by suppression of the NF-κB pathway, could theoretically enhance the effects of nonsteroidal anti-inflammatory drugs (NSAIDs) or corticosteroids, possibly leading to additive effects or increased risk of adverse outcomes [127]. Similarly, its antioxidant effects via Nrf2 activation may interact with chemotherapeutic agents or other redox-modulating drugs, although such interactions remain speculative due to limited in vivo data.

Of particular concern are potential interactions with sedatives or anxiolytics. Since *C. asiatica* exhibits mild anxiolytic effects and modulates neurotransmitter pathways, it may exert additive effects when combined with benzodiazepines, barbiturates, or other central nervous system depressants [128]. Although human data are lacking, clinicians should remain vigilant when co-administering *C. asiatica* with psychotropic medications.

Given these considerations, healthcare providers and patients should approach the concurrent use of *C. asiatica* and pharmaceutical drugs cautiously. While no major contraindications have been formally established, pharmacokinetic and toxicological studies are needed to establish a more comprehensive safety profile and elucidate herb–drug interaction risks.

### 7.6. Regulatory Perspectives and Quality Control

The global interest in *C. asiatica* as a therapeutic and cosmeceutical agent has necessitated the development of standardized regulatory frameworks to ensure product safety, efficacy, and consistency. Despite its inclusion in several national pharmacopoeias, including the Ayurvedic Pharmacopoeia of India [129] and the Chinese Pharmacopoeia [3], there is a lack of harmonized international standards for extract preparation, phytochemical content, and clinical indications.

In the European Union and North America, *C. asiatica* is predominantly regulated as a dietary supplement or traditional herbal remedy [130,131]. Under these classifications, manufacturers are required to ensure product quality and safety but are not obligated to demonstrate efficacy through clinical trials. This regulatory gap has led to substantial variation in product composition and bioactivity across different markets. In contrast, countries such as South Korea and Thailand have implemented stricter guidelines, often requiring standardization to specific triterpenoid markers, such as asiaticoside and madecassoside [132,133].

Quality control remains a major challenge. Variation in plant genotype, growing conditions, harvest timing, and extraction methods contributes to inconsistencies in the final product. The use of validated analytical techniques such as HPLC, LC-MS, and NMR is essential for ensuring chemical consistency. Pharmacopoeial monographs increasingly emphasize the need for standardized extracts containing defined quantities of active compounds, typically not less than 1% asiaticoside and 0.5% madecassoside [3,129].

Moreover, GACP, Good Manufacturing Practices (GMP), and green extraction principles are being adopted to align the industry with sustainability goals and ensure the traceability of raw materials [22,72,75]. Compliance with these guidelines is critical not only for regulatory approval but also for maintaining consumer trust in the safety and efficacy of *C. asiatica*-based products. In summary, the effective integration of *C. asiatica* into evidence-based healthcare systems will depend on robust regulatory oversight, stringent quality assurance, and continued scientific validation. Standardized extract formulations backed by clinical data will be essential for future pharmacological development and commercialization.

## 8. Healthcare and Commercial Applications

In pharmaceuticals, *C. asiatica* is gaining prominence for its efficacy in treating skin and connective tissue disorders. It has been included in topical formulations used in wound care, burn management, and post-surgical healing [95,108,112]. Notably, madecassoside-containing creams and gels have been clinically validated for their ability to accelerate re-epithelialization, enhance angiogenesis, and improve scar appearance [95,108,112]. A well-documented example is the use of madecassoside-enriched preparations in diabetic wound care, where patients treated with these formulations demonstrated faster healing rates, improved tissue regeneration, and reduced inflammatory response compared to standard care [134,135]. These outcomes are attributed to the modulation of TGF-β signaling, stimulation of fibroblast proliferation, and promotion of collagen synthesis mechanisms supported by both preclinical and clinical studies [31,61,62].

In the nutraceutical domain, *C. asiatica* is marketed globally as a natural cognitive enhancer and anti-stress agent. Its adaptogenic profile makes it a key component in supplements designed to support mental clarity, memory retention, and resistance to stress-induced fatigue [105,110,111]. Formulations combining *C. asiatica* with phosphatidylserine, ginkgo biloba, or bacopa monnieri are increasingly popular in brain health supplements targeting aging populations and high-performance professionals [136,137]. Market analysts project significant growth in this sector, with *C. asiatica*-based cognitive and mood-enhancing supplements contributing to the USD 300+ billion global nutraceutical industry [138,139].

In terms of commercial applications, the cosmeceutical of *C. asiatica* has become a mainstay in anti-aging and skin-repair formulations. Its inclusion in serums, creams, and masks is backed by its ability to stimulate collagen production, improve skin elasticity, and reduce transepidermal water loss [113,114,115]. The triterpenoids in *C. asiatica* modulate matrix metalloproteinase (MMP) activity and support extracellular matrix integrity, both of which are critical in combating signs of photoaging and maintaining dermal structure [38,140]. Madecassoside and asiaticoside have been patented for their roles in soothing sensitive skin, preventing stretch marks, and enhancing post-laser skin recovery [141]. Brands such as La Roche-Posay and Innisfree have commercialized *C. asiatica*-based lines, reflecting the herb’s successful integration into global skincare markets. Current cosmeceutical trends also focus on natural and “green beauty” ingredients, which have further increased demand for botanicals like *C. asiatica*, known for both efficacy and ecological sustainability. Table 3 summarizes the clinical and commercial applications of *C. asiatica* across healthcare sectors.

The convergence of biotechnology and herbal medicine has opened new avenues for *C. asiatica* in biotherapeutic development. Novel delivery systems such as liposomes, nanofibers, and hydrogels are being explored to improve the bioavailability of their active compounds and enhance their therapeutic precision [141,142,143,144]. Furthermore, patented nano-emulsion formulations are under investigation for enhanced skin penetration and sustained release of madecassoside, indicating the plant’s growing relevance in advanced dermatological therapies.

Importantly, *C. asiatica* occupies a unique position bridging herbal biotherapeutics and allopathic chemotherapeutics. In herbal contexts, its crude extracts and phytoconstituent-rich fractions are utilized for holistic treatment strategies. In contrast, purified triterpenoids and phytosterols are being investigated within allopathic paradigms for targeted interventions, including neurodegenerative diseases, vascular protection, and metabolic regulation [145]. This dual utility exemplifies the adaptability of *C. asiatica* across therapeutic frameworks, aligning with the growing emphasis on integrative and personalized medicine.

In summary, as innovation in formulation technologies continues and regulatory clarity improves, *C. asiatica* is expected to play an increasingly prominent role in global healthcare. Its compatibility with traditional wisdom and modern scientific validation positions *C. asiatica* not only as a phytomedicinal agent of the present but also as a cornerstone for the future of botanical therapeutics.

## 9. Future Prospects

The expanding therapeutic interest in *C. asiatica* offers fertile ground for innovation across scientific, clinical, and technological domains. One of the most promising frontiers lies in the application of artificial intelligence (AI) and machine learning (ML) for compound discovery, activity prediction, and formulation optimization. Computational models trained on large-scale phytochemical databases and bioactivity datasets can accelerate the identification of novel bioactive compounds within *C. asiatica*, predict molecular targets, and simulate pharmacokinetic behaviors [146,147]. These AI-driven approaches have the potential to reduce time and cost in preclinical development and guide rational extract standardization by prioritizing the most pharmacologically relevant constituents.

Simultaneously, advances in pharmacogenomics may revolutionize the personalized application of *C. asiatica*-based therapeutics [148]. Genetic variability in drug metabolism enzymes, transporters, and receptor profiles can influence individual responses to phytomedicine. By leveraging genomic screening tools, clinicians may eventually tailor *C. asiatica* dosing and formulation to maximize efficacy and minimize adverse effects, especially in populations with metabolic or hepatic vulnerabilities. Such precision-based approaches could prove transformative in neurodegenerative disease management, dermatological care, and integrative psychiatry, where interindividual variability often complicates outcomes.

Another critical area for expansion is the systematic integration of *C. asiatica* into national clinical practice guidelines and public health frameworks. Its potential applications in skin wound care, cognitive decline prevention, and metabolic syndrome align with priority areas in both developed and resource-limited healthcare settings. However, this integration will require robust, high-quality clinical evidence, harmonized product regulation, and ongoing pharmacovigilance. Collaboration between academia, regulatory agencies, and industry stakeholders is vital to promote translational research and ensure that *C. asiatica*-based products meet global standards for efficacy, safety, and accessibility. Finally, sustainable sourcing and green extraction must be embedded in the future roadmap to ensure environmental stewardship and ethical trade. Bioengineering, vertical farming, and synthetic biology approaches may also offer scalable alternatives to wild harvesting, preserving biodiversity while supporting consistent phytochemical quality [149,150,151].

## 10. Conclusions

*Centella asiatica* or *C. asiatica* stands as a botanically rich and pharmacologically versatile plant with proven therapeutic promise across dermatological, neurocognitive, and anti-inflammatory domains. Despite centuries of traditional use and a growing body of mechanistic and clinical data, several critical gaps remain. Chief among them is the lack of standardized extracts and consensus on dosing regimens, which has limited reproducibility across trials and hindered regulatory acceptance. Most existing studies are constrained by small sample sizes, short durations, and variable product quality, making large-scale, randomized controlled trials an urgent priority. Further, although the molecular mechanisms of *C. asiatica*, including its effects on NF-κB, Nrf2, MAPK, and PI3K/Akt pathways, are increasingly well-characterized, the pharmacokinetics of its key bioactive remain poorly understood. Addressing these gaps through targeted pharmacological studies, advanced drug delivery systems, and integration with modern technologies such as AI and genomics will be critical for advancing *C. asiatica* from a traditional remedy to a rigorously validated, globally accessible therapeutic. In conclusion, the future of *C. asiatica* lies not only in its pharmacological potential but in the scientific rigor, technological innovation, and collaborative frameworks applied to its development. With strategic investments in research, regulation, and sustainability, *C. asiatica* may evolve into a cornerstone botanical in 21st-century integrative and personalized medicine.

## Figures and Tables

**Table 1:** Comparative analysis of extraction techniques for *Centella asiatica*.

Method	Yield	Selectivity	Energy Consumption	Eco-Friendliness	Scale-Up Feasibility
SFE [71,72]	High	High	Moderate	Excellent	Moderate
MAE [73,74]	Moderate to High	Moderate	High	Good	High
UAE [75,76]	High	Moderate	Low	Very Good	High
EAE [77,78,79]	Moderate	High	Low	Excellent	Moderate
PLE [79,80]	High	Moderate to High	Moderate	Good	High

EAE, enzyme-assisted extraction; MAE, microwave-assisted extraction; PLE, pressurized liquid extraction; SFE, supercritical fluid extraction; UAE, ultrasound-assisted extraction.

**Table 2:** Dosage and pharmacokinetics of *C. asiatica* extracts and key bioactive compounds.

Parameter	Extract/Compound	Dosage Range	Pharmacokinetic Characteristics	Clinical Notes
Cognitive enhancement [107,110,111]	Standardized extract (whole plant)	300–750 mg/day orally	Low oral bioavailability, limited systemic exposure, and unclear metabolites	Administered in divided doses; effects observed after 4–12 weeks of use
Wound healing (topical) [95,108,112]	Madecassoside or asiaticoside cream	1% *w*/*w*, applied twice daily	Localized dermal penetration; avoids first-pass metabolism	Demonstrated enhanced collagen synthesis and reduced scar formation in diabetic ulcers
Skin repair/ Cosmeceutical [113,114,115]	Triterpenoid-enriched cream/gel	0.5–1% *w*/*w* topical application	Improved penetration with nanoemulsion/ liposomes	Used in anti-aging, post-laser recovery, and acne-prone skin
Anti-inflammatory (oral) [83,116,117]	TECA (Titrated Extract of *C. asiatica*)	60–120 mg/day (standardized)	Rapid first-pass metabolism, plasma half-life < 2 h	Variable responses: chronic dosing recommended for vascular and inflammatory conditions
Bioavailability studies [118,119]	Asiatic acid, madecassic acid	30–50 mg/kg (preclinical)	Poor absorption, high hepatic metabolism, and low peak plasma levels	Nanocarrier and phytosome formulations under development to enhance systemic delivery
Topical formulations [120,121]	Whole plant extract	Depends on the formulation matrix	Primarily local activity, systemic absorption is minimal	High safety margin; limited systemic toxicity

Data based on human clinical studies and preclinical pharmacokinetic investigations (e.g., rat/mouse models). TECA refers to a titrated extract standardized to 40% asiaticoside, 30% madecassoside, 30% asiatic and madecassic acids. Oral use in humans typically suffers from low bioavailability due to poor water solubility and rapid metabolism. Topical use avoids systemic metabolism and has shown superior outcomes in dermatological applications.

**Table 3:** Clinical and commercial applications of *Centella asiatica* across healthcare sectors.

Application Domain	Therapeutic Indication	Formulation Type	Clinical Evidence	Commercial Examples/Products
Pharmaceutical	Wound healing (e.g., diabetic ulcers)	Creams, ointments, and hydrogels with madecassoside	RCTs show improved re-epithelialization, collagen synthesis, and reduced scarring	Madecassol^®^, Emdecassol, Tiger Balm
	Venous insufficiency	Oral tablets/topical gels	Clinical trials report reduced edema and capillary permeability	Centelase, Titrated Extract of *C. asiatica* (TECA)
	Anti-inflammatory and neuroprotection	Standardized extracts	Preclinical and limited clinical trials show NF-κB modulation and antioxidant effects	Under research—potential NCE (new chemical entity)
Nutraceutical	Cognitive support, stress adaptation	Capsules, soft gels, nootropic blends	Systematic reviews suggest modest improvement in memory and anxiety	NeuroGain^®^, Himalaya Mentat, Focus Factor
	General wellness and anti-aging	Oral powders, adaptogenic blends	Observational studies support antioxidant effects; clinical trials are ongoing	Nature’s Answer, Organic India
Cosmeceutical	Skin repair and anti-aging	Creams, serums, masks with asiaticoside/madecassoside	Controlled studies confirm improved skin texture, elasticity, and hydration	La Roche-Posay Cicaplast, Innisfree Cica Line
	Acne, sensitive skin	Gels, post-laser treatments	Meta-analysis shows a reduction in acne lesions, redness, and skin irritation	SNP Cica Repair, Etude House Madecassoside Gel

## Data Availability

Not applicable.

## Abbreviations

The following abbreviations are used in this manuscript:

AFLP	Amplified fragment length polymorphism
BDNF	Brain-derived neurotrophic factor
CAT	Catalase
CBD	Convention on Biological Diversity
CO_2_	Carbon dioxide
COX-2	Cyclooxygenase-2
EAE	Enzyme-assisted extraction
ERK1/2	Extracellular signal-regulated kinases 1 or 2
FGF	Fibroblast growth factor
GACP	Good Agricultural and Collection Practices
GC-MS	Gas chromatography-Mass spectrometry
GMP	Good Manufacturing Practices
HO-1	Heme oxygenase-1
HPLC	High-performance liquid chromatography
IL-1β	Interleukin-1β
iNOS	Inducible nitric oxide synthase
IUCN	International Union for Conservation of Nature
JNK	c-Jun N-terminal kinases
LC-MS	Liquid chromatography-Mass spectrometry
MAE	Microwave-assisted extraction
MAPK	Mitogen-activated protein kinases
MMP	Matrix metalloproteinase
MS	Mass spectrometry
mTOR	Mammalian target of rapamycin
NF-κB	Nuclear factor kappa B
NMR	Nuclear magnetic resonance
Nrf2	Nuclear factor erythroid 2-related factor 2
NSAIDs	Nonsteroidal anti-inflammatory drugs
OH	Hydroxyl
PI3K	Phosphatidylinositol 3-kinase
PI3K/Akt	Phosphatidylinositol 3-kinase/protein kinase B
PLE	Pressurized liquid extraction
RAPD	Randomly amplified polymorphic DNA
ROS	Reactive oxygen species
SFE	Supercritical fluid extraction
SOD	Superoxide dismutase
SSR	Simple sequence repeats
TGF-β1	Transforming growth factor beta 1
TNF-α	Tumor necrosis factor alpha
UAE	Ultrasound-assisted extraction
UPLC	ultra-performance liquid chromatography
VEGF	Vascular endothelial growth factor
WHO	World Health Organization
